# International *Salmonella* Typhimurium DT104 Infections, 1992–2001

**DOI:** 10.3201/eid1106.041017

**Published:** 2005-06

**Authors:** Morten Helms, Steen Ethelberg, Kåre Mølbak, the DT104 study group

**Affiliations:** * Statens Serum Institut, Copenhagen, Denmark

**Keywords:** multidrug resistance, Salmonella, survey, S. Typhimurium, DT104

## Abstract

The incidence of multidrug-resistant (MDR) *Salmonella* Typhimurium infections in humans, and in particular MDR definitive phage type 104 (DT104), has increased substantially in many countries in the last 2 decades, often associated with increased illness. To examine the magnitude of this problem, a survey was conducted among countries with available antimicrobial resistance or phage typing surveillance data. A total of 29, primarily industrialized, countries participated in the survey, which covered the years 1992–2001. Overall, the incidence of MDR *S*. Typhimurium and DT104 increased continuously during this period, although the problem affected primarily Europe and North America. The increase appeared to have peaked in the United Kingdom but not in other countries. Also, the incidence of quinolone-resistant *S*. Typhimurium was increasing. This survey implies that MDR *S*. Typhimurium constitutes an increasing public health problem in large parts of the world and emphasizes the importance of surveillance and control programs.

Infections with nontyphoidal *Salmonella* have increased during the last 3–4 decades, and although a decrease has been reported over the last decade, *Salmonella* infections continue to be a major public health concern in many countries (1–3). These salmonellae are zoonotic, and the infections are generally foodborne. Although a large number of *Salmonella* serotypes exist, the overall increase in the number of infections is of relatively few emerging serotypes and phage types. Over periods of several years, certain *Salmonella* types have risen and (sometimes) fallen within large geographic regions. These meta-outbreaks are facilitated through the acquisition, by specific types, of new traits that make them well adapted to spread, as well as through changes to human society, seen, for instance, with modern intensified farming and food production methods and global trade with live breeder animals (2,4,5). Two prominent examples are the international spread of *S*. Enteritidis infections through hens eggs (2,6) and the emergence over the last 2 decades of multidrug-resistant (MDR) *Salmonella* Typhimurium definitive phage type 104 (DT104).

Nontyphoidal *Salmonella* causes mild to severe, including life-threatening, infections. One study estimated that 600 deaths occur per year in the United States alone due to infections with nontyphoidal *Salmonella* serotypes (7). A recent study in Denmark showed that infection with nontyphoidal *Salmonella* was associated with a 2.5-fold increased risk for death within 1 year of infection compared with a matched sample from the general Danish population (8). Salmonellae resistant to antimicrobial drugs appear to pose a particular health risk. Thus, several studies have indicated that infection with salmonellae resistant to ≥1 antimicrobial drugs is associated with increased risk for hospitalization, invasive illness, and death (9–15).

In general, antimicrobial drug resistance occurs frequently in zoonotic salmonellae and is largely promoted by using antimicrobial drugs in food animals (4,16–18). *S*. Typhimurium DT104 is commonly resistant to 5 drugs: ampicillin, chloramphenicol, streptomycin, sulfonamides, and tetracycline (R-type ACSSuT). *S*. Typhimurium DT104 was first isolated in the early 1980s in the United Kingdom and later became endemic in bovine animals, from where it spread to the whole food animal production in that country (5,18). Throughout the 1990s, it spread to other parts of the world, and it is now a common *Salmonella* type in many countries, including the United States, the United Kingdom, Germany, and France (2,19–22). DT104 is common in a broad range of food animals, such as poultry, pigs, and sheep (23). This phage type has become a matter of concern because of its rapid international dissemination in the 1990s and its ability to readily acquire additional resistance traits to other, clinically important antimicrobial drug classes, such as quinolones, trimethoprim, and cephalosporins.

A global survey of salmonellosis and *Salmonella* serotyping was published in 2002 (24). However, relatively little information has been compiled on the global spread of DT104 and MDR *S*. Typhimurium (23). Therefore, we have conducted a survey to describe the pandemic of DT104 and MDR *S*. Typhimurium.

## Methods

### Participating Countries

The survey addressed information on antimicrobial drug resistance testing, phage typing, or both. Since most countries do not routinely apply these typing methods, the questionnaire was not simply sent to all World Health Organization (WHO) member states. Instead, an invitation to participate in the survey was distributed through the WHO Global Salm-Surv (GSS) network (25) and directly to all Enter-Net (26) network countries, plus a group of large countries known or assumed to have resistance testing or phage typing as an integrated part of their national *Salmonella* surveillance system.

A total of 52 questionnaires were sent out in June and July 2002. Of these, 44 were sent directly to countries known or assumed to have resistance testing or phage typing, and 8 were sent out to member states responding to the GSS invitation. Of the 52 questionnaires, 32 were sent to countries in the European region, 6 to the American Region, 7 to the Asian Region, 5 to the African Region, and 2 to the Oceania Region. Country names and names of geographic regions and subregions are used as described in the United Nations classification system (27).

### The Questionnaire

Each country was requested to give information on the total annual number of laboratory-verified episodes of nontyphoidal salmonellosis, *S*. Typhimurium, MDR *S*. Typhimurium, and *S*. Typhimurium DT104 from 1992 to 2001. The participating countries were also asked to give details on whether the reported number of *Salmonella* isolates that formed the basis for further phage typing or antimicrobial drug susceptibility testing included all national isolates or if they were a subset of isolates. If only a subset of isolates were tested, the countries were asked to state the proportion of *S*. Typhimurium strains tested and what the criteria for choosing the isolates were. We also asked participants to describe methods used for antimicrobial drug resistance testing and the drugs included in their tests.

Multidrug-resistance was defined as isolates being resistant or intermediately susceptible towards ≥4 separate classes of drugs. This group includes isolates of R-type ACSSuT, the so-called classical penta-resistant phenotype. When information was available, participants were asked to give the numbers of isolates exhibiting R-types extending the ACSSuT-complex and the number of isolates resistant to clinically relevant antimicrobial drugs (quinolones, trimethoprim, and cephalosporins).

In countries where only a subset of *Salmonella* isolates had been submitted for phage typing, antimicrobial drug-resistance testing, or both, the total number of DT104 or MDR *S*. Typhimurium isolates was extrapolated from the reported numbers of isolates and the proportion of tested isolates.

## Results

**Figure 1:**
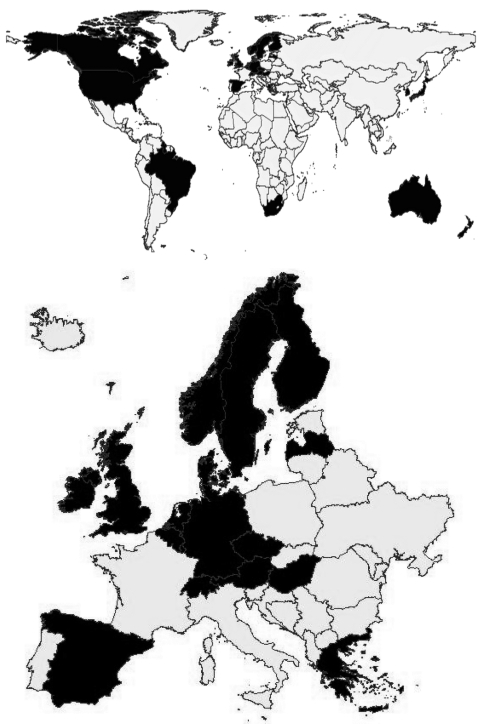
Participating countries in the survey of multidrug-resistant *Salmonella enterica* serotype Typhimurium, 1992–2001, internationally (A) and in Europe (B).

The questionnaire was sent to 52 countries, and a completed questionnaire was received from 29, a response rate of 56% (Figure 1). Of the 52 invited countries, 23 were members of or affiliated with Enter-Net; from these a positive feedback was received from 20 (87%). These countries were Australia, Austria, Belgium, Canada, Denmark, England and Wales, Finland, Germany, Greece, Ireland, Japan, Luxembourg, the Netherlands, New Zealand, Norway, Scotland, South Africa, Spain, Sweden, and Switzerland. The 8 countries and 1 regional center (31% response rate) that participated in the survey that were not associated with Enter-Net when data were collected were Brazil, the Czech Republic, Hungary, Israel, Latvia, Malta, Republic of South Korea, United States, and the Caribbean Epidemiology Centre (CAREC), a regional center that represents 21 countries in the Caribbean (Anguilla, Antigua and Barbuda, Aruba, Bahamas, Barbados, Belize, Bermuda, British Virgin Islands, Cayman Islands, Dominica, Grenada, Guyana, Jamaica, Montserrat, Netherlands Antilles, St. Kitts and Nevis, St. Lucia, St. Vincent and the Grenadines, Suriname, Trinidad and Tobago, and Turks and Caicos). For the purpose of this study, CAREC was treated as 1 unit. The 29 participating countries had a total population of 1.028 billion in 2001.

### Surveillance

The proportion of *Salmonella* isolates forwarded from local health laboratories to national or regional institutions varied from 5% to 100%. None of the participating countries restricted the submission of isolates to strains from selected patients, e.g., in case of septicemia or outbreak situations. All 29 participating countries performed serotyping on 85% to 100% of *Salmonella* isolates received at the national reference laboratory. Table 1 shows the number of nontyphoidal *Salmonella* isolates and the proportion hereof that were *S*. Typhimurium in each country from 1992 to 2001 presented as 2-year intervals. The table also shows the estimated incidence of laboratory-confirmed cases of *S*. Typhimurium in 2001. Throughout the study period, the proportion of *S*. Typhimurium among nontyphoidal salmonellae has been relatively stable. In 1992, 16% of isolates were *S*. Typhimurium, compared to 17% in 2001. However, large differences between countries and variations from year to year within each country made comparison difficult. Nevertheless, some trends emerged when the countries were aggregated into geographic regions. In the Caribbean region, South America, Eastern Asia, and Europe, a general decrease in the proportion of *S*. Typhimurium was observed. In North America, on the other hand, the situation has remained relatively stable, whereas both Australia and New Zealand have seen an increase in the number and proportion of *S*. Typhimurium cases from 1992 to 2001.

**Table 1:** Number of patients with culture-confirmed, nontyphoidal *Salmonella* infection and percentage *S*. Typhimurium, international DT104 survey, 1992–2001

Subregion/country	Nontyphoidal *Salmonella* infections (% *S*. Typhimurium)					
	1992–1993	1994–1995	1996–1997	1998–1999	2000–2001	2001 pop. (IST)*
Northern Europe						
England + Wales	62,005 (16.4)	59,725 (20.5)	61,579 (16.8)	41,260 (13.2)	31,309 (15.1)	53 (3.9)
Ireland				1,152 (65.6)	1,147 (36.5)	3.8 (3.9)
Scotland	5,911 (20.1)	6,076 (21.6)	6,615 (19.8)	3,988 (16.0)	3,291 (17.8)	5.1 (5.0)
Denmark	7,184 (34.5)	7,923 (28.0)	8,273 (21.1)	7,148 (17.7)	5,257 (19.5)	5.4 (10.9)
Finland†	6,770 (13.0)	6,070 (14.1)	5,615 (16.9)	5,536 (12.4)	5,358 (10.4)	5.2 (5.0)
Latvia	1,048 (54.2)	1,965 (41.9)	1,393 (29.0)	1,830 (23.1)	1,664 (9.3)	2.4 (2.8)
Norway†	2,181 (18.1)	2,386 (13.3)	2,632 (16.4)	2,933 (14.9)	3,394 (13.5)	4.5 (4.9)
Sweden†	5,296 (17.5)	11,249 (13.3)	10,479 (11.5)	11,398 (13.8)	11,119 (14.1)	8.9 (8.3)
Western Europe						
Austria	11,337 (10.0)	19,790 (5.5)	18,278 (5.2)	16,918 (4.7)	15,135 (5.6)	8.3 (5.6)
Belgium	21,231 (34.7)	22,048 (31.9)	26,247 (26.2)	30,288 (21.7)	25,153 (20.6)	10.3 (23.0)
Germany‡	335,813 (17.1)	248,507 (21.9)	215,524 (26.4)	183,697 (27.1)	163,327 (26.2)	82.2 (8.4)
Luxembourg	490 (27.8)	447 (17.4)	555 (22.5)	652 (22.9)	701 (23.8)	0.44 (19.8)
Netherlands	5,388 (36.2)	5,955 (26.2)	5,445 (32.8)	4,393 (31.1)	4,141 (31.8)	16.0 (6.9)
Switzerland§					2,374 (10.2)	7.2
Eastern Europe						
Czech Republic	85,097 (6.8)	103,342 (2.6)	88,421 (2.3)	95,323 (2.1)	73,529 (1.9)	10.3 (5.3)
Hungary		14,138 (15.0)	11,398 (12.6)	8,188 (20.2)	8,480 (10.7)	10.0 (5.5)
Southern Europe						
Greece	1,198 (13.4)	1,514 (14.5)	1,315 (18.2)	1,091 (24.3)	1,947 (20.5)	10.9 (13.4)
Malta	546 (20.1)	518 (26.6)	236 (19.1)	390 (3.6)	135 (34.8)	0.40 (9.0)
Spain¶	6,374 (25.9)	7,188 (29.0)	8,847 (32.9)	10,806 (25.6)	13,379 (21.1)	39.8 (3.5)
Israel#	15,337 (10.7)	13,674 (8.1)	10,168 (27.2)	9,875 (16.8)	9,353 (15.0)	6.4 (11.4)
North America						
Canada	14,863 (19.2)	13,645 (20.3)	14,596 (23.0)	16,028 (21.0	12,493 (20.9)	31.0 (4.9)
USA	71,605 (22.6)	78,723 (22.5)	73,643 (25.3)	66,753 (25.3)	63,697 (22.1)	281.4 (2.6)
Caribbean region			687 (22.1)	786 (22.8)	379 (10.6)	7.1 (0.3)
Brazil	2,276 (10.5)	7,500 (2.7)	11,832 (1.5)	10,511 (3.8)	15,455 (4.8)	171.8 (0.3)
South Africa					1,341 (51.3)	43.6 (1.2)
Eastern Asia						
Japan	18,385 (8.4)	22,406 (6.1)	27,260 (2.8)	23,359 (3.2)	11,889 (5.3)	127.4 (0.2)
Republic of Korea	1,918 (37.4)	1,567 (32.0)	2,314 (18.7)	3,548 (15.9)	2,404 (11.3)	48.8 (0.3)
Oceania						
Australia	9,496 (28.1)	11,794 (35.2)	13,229 (40.2)	15,104 (35.2)	13,053 (38.9)	19.4 (13.7)
New Zealand	2,579 (56.2)	3,137 (58.3)	2,658 (56.6)	4,472 (63.1)	4,517 (64.7	3.9 (42.7)

*pop., population ×10^6^; IST, incidence of *S*. Typhimurium per 10^5^ population. †Most patients in these countries were infected abroad. ‡Data on *S*. Typhimurium and incidence are based on data from the new federal states of Germany and the city of Berlin. §Submission of strains for serotyping at the central laboratory was not compulsory in Switzerland at the time of data collection. As a result an incidence rate for Switzerland is not presented. ¶Incidence is based on data from 2000. #According to the United Nations classification, Israel belongs to the western Asian region. In this study, Israel has been grouped with southern European countries.

### Phage Typing

In 1992, phage typing was carried out in 6 of the participating countries; by 2001, this number had risen to 22. Five countries (Brazil, Japan, Norway, Switzerland, and the United States) only performed phage typing when an MDR strain was found or in outbreak situations. Although not all countries performed phage typing in accordance with the Colindale Scheme (28), the countries using different standards provided information that allowed for comparison with results obtained using the Colindale Scheme (29–31).

**Figure 2:**
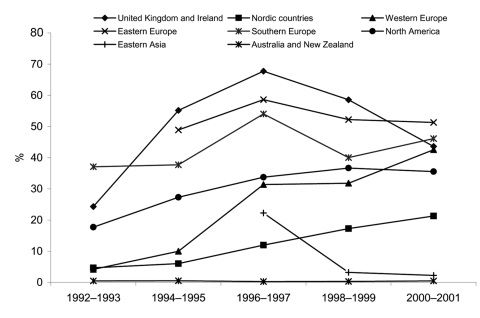
*Salmonella enterica* serovar Typhimurium DT104 as percentage of all *S*. Typhimurium in 8 world regions, 1992–2001. Only countries that had data available for 2 or more 2-year periods are included: United Kingdom and Ireland: England and Wales, Scotland, Ireland; Scandinavia: Denmark, Finland, Norway, Sweden; Western Europe: Austria, Germany, and the Netherlands; Eastern Europe: Czech Republic and Hungary; Southern Europe: Spain and Israel; North America: Canada, United States; Eastern Asia: the Republic of Korea; Oceania: Australia and New Zealand.

The proportion of *S*. Typhimurium strains that were DT104 and the proportion thereof that were found to be MDR are shown for each country in Table 2. In general, the incidence and proportion of DT104 increased throughout the period. In 1992, 8.7% of *S*. Typhimurium isolates were DT104, but in 2001 this proportion had increased to 33%. Again, large regional differences occurred. In the United Kingdom, the incidence peaked in 1996 and then decreased. In most other European countries and North America, the relative numbers of DT104 strains had increased throughout the period. In Australia and New Zealand, few DT104 isolates (0.7%) were seen among *S*. Typhimurium in 2001. The proportion of *S*. Typhimurium strains that were DT104 is depicted in Figure 2 (the countries are aggregated into 8 regions).

**Table 2:** Number (regional level) and proportion (%) of multidrug resistance and definitive phage type 104 (DT104) in *Salmonella* Typhimurium in 29 countries participating in the international DT104 survey*

Subregion/country	% MDR, % DT104 (% DT104 that are MDR)				
	1992–1993	1994–1995	1996–1997	1998–1999	2000–2001
Northern Europe†	479; 2,802; 2,143†	910; 7,590; 6,629	1,190; 8,345; 7,520	1,729; 4,698; 2,969	1,380; 3,270; 824
England + Wales	NA, 22.6 (75.5)	NA, 54.5 (87.0)	NA, 67.5 (94.0)	NA, 57.1 (92.0)	NA, 42.3 (NA)
Ireland	40.3, 38.9 (82.5)	66.8, 61.2 (90.9)	76.1, 70.0 (92.6)	70.7, 65.4 (92.3)	63.3, 45.2 (90.6)
Scotland				75.0, 63.1 (73.6)	79.7, 56.3 (89.8)
Denmark		1.5, 1.5 (69.2)	5.0, 3.0 (80.8)	21.9, 15.8 (93.5)	23.1, 12.7 (91.5)
Finland	NA, 4.7 (61.0)	NA, 10.5 (83.3)	NA, 11.5 (78.0)	NA, 11.4 (87.2)	34.8, 27.9 (95.5)
Latvia				79.7, NA (NA)	63.2, NA (NA)
Norway			24.5, NA (NA)	22.0, 12.6 (100.0)	32.0, 24.0 (95.5)
Sweden			NA, 25.3 (NA)	NA, 22.3 (NA)	NA, 23.7 (NA)
Western Europe†	2,807; 885; 609†	5,242; 1,944; 1,519	8,407; 6,346; 5,308	7,782; 5,347; 4,539	10,048; 7,626; 6,377
Austria	NA, 17.0 (NA)	NA, 14.6 (NA)	13.7, 32.7 (70.6)	13.1, 28.9 (67.5)	35.8, 29.6 (82.1)
Belgium					39.6, 27.0 (79.9)
Germany	14.3, 3.1 (89.3)	30.2, 9.2 (91.7)	44.3, 32.1 (87.1)	49.0, 32.1 (86.2)	57.1, 44.0 (84.7)
Luxembourg	11.0, NA (NA)	26.9, NA (NA)	43.2, NA (NA)	55.0, NA (NA)	58.1, NA (NA)
Netherlands	10.3, 6.7 (81.5)	8.9, 15.3 (43.1)	26.3, 23.5 (78.1)	29.5, 29.6 (79.8)	33.9, 37.2 (80.8)
Switzerland					48.8, 28.5 (100.0)
Eastern Europe†		NA; 1,034; NA	117; 2,020; 117	778; 1,902; 732	889; 1,182; 764
Czech Republic			5.8, 57.3 (10.1)	11.7, 43.9 (26.1)	28.5, 47.1 (54.6)
Hungary		NA, 48.9 (NA)	NA, 60.4 (NA)	33.0, 62.2 (84.0)	54.1, 57.7 (77.3)
Southern Europe†	121; 612; NA†	166; 1,206; NA	1,126; 3,070; 222	112; 1,771; NA	1,360; 1,296; 674
Greece	18.1, NA (NA)	61.6, NA (NA)	44.8, NA (NA)	39.2, NA (NA)	26.3, NA (NA)
Malta	83.6, NA (NA)	22.5, NA (NA)	20.0, NA (NA)	57.1, NA (NA)	4.3, NA (NA)
Spain	NA, 37.1 (NA)	NA, 26.0 (NA)	34.6, 20.2 (80.4)	NA, 23.4 (NA)	45.0, 18.3 (65.0)
Israel‡		NA, 59.5 (NA)	NA, 89.6 (NA)	NA, 67.7 (NA)	85.6, 73.9 (97.7)
North America†	NA; 506; NA†	3,507; 755; 237	9,045; 4,209; 3,265	8,315; 4,466; 3,528	6,748; 930; 785
Canada	NA, 17.7 (NA)	NA, 27.3 (42.5)	16.2, 46.1 (51.8)	44.1, 43.8 (82.3)	47.8, 35.5 (84.4)
USA		19.8, NA (NA)	45.7, 29.1 (92.6)	40.5, 34.0 (77.4)	39.0, NA (NA)
Caribbean region†			0; NA; NA	0; NA; NA	0; NA; NA
Caribbean			0.0, NA (NA)	0.0, NA (NA)	0.0, NA (NA)
South America†		1; NA; NA	7; NA; NA	52; NA; NA	97; NA; NA
Brazil		0.5, NA (NA)	4.0, NA (NA)	13.0, NA (NA)	13.0, NA (NA)
Southern Africa†					74; NA; NA
South Africa					11.2, NA (NA)
Eastern Asia†	NA; 32; 32†	NA; 43; 43	62; 112; 111	44; 121; 121	37; 68; 68
Japan	NA, 2.1 (100.0)	NA, 3.1 (100.0)	NA, 8.8 (100.0)	NA, 13.7 (100.0)	NA, 9.8 (100.0)
Republic of Korea			14.3, 22.2 (97.8)	7.8, 3.2 (100.0)	13.7, 2.2 (100.0)
Oceania†	100; 13; 12†	114; 9; 8	80; 4; 4	95; 16; 16	163; 38; 27
Australia	3.8, 0.1 (100.0)	2.7, NA (NA)	1.5, NA (NA)	1.8, 0.2 (100.0)	3.2, 0.7 (68.6)
New Zealand	NA, 0.8 (91.7)	NA, 0.5 (88.9)	NA, 0.3 (100.0)	NA, 0.4 (100.0)	NA, 0.1 (100.0)

*MDR, multidrug-resistant; NA, data not available. The table also shows the % MDR DT104 of all DT104 strains. †No. of MDR; no. of DT104; and no. of DT104 that are MDR. ‡According to the United Nations classification, Israel belongs to the western Asian region. In this study, Israel has been grouped with southern European countries.

### Antimicrobial Drug Resistance

Antimicrobial susceptibility testing became an increasingly common constituent of the surveillance during the study period. In 1992, susceptibility testing was performed in 14 of the 29 participating countries and in all but one in 2000. All countries routinely tested a minimum of 6 antimicrobial drugs, all belonging to different drug classes. The majority of countries (73%) used disc diffusion testing according to NCCLS standards. Listed by frequency, the most common antimicrobial drug classes included in the test panel were (fluoro)quinolones, broad-spectrum penicillins, phenicols, aminoglycosides, tetracyclines, cephalosporins, sulfonamides, and trimethoprim. In 21 countries, antimicrobial susceptibility testing was performed independently of phage typing results, but in 2 countries (Finland until 1999 and New Zealand), testing was only performed on strains found to be DT104.

**Figure 3:**
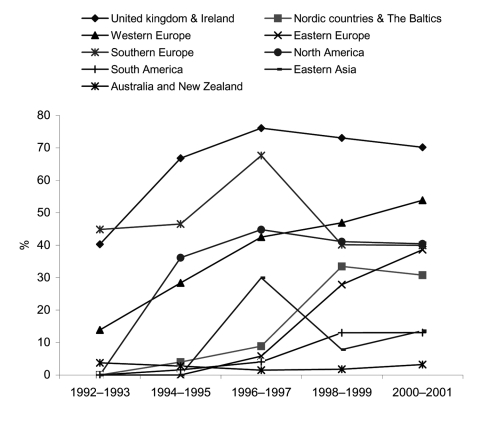
Multidrug-resistant *Salmonella enterica* serovar Typhimurium as a percentage of all *S*. Typhimurium in 9 world regions, 1992–2001. Only countries that had data available for 2 or more 2-year periods are included: United Kingdom and Ireland: Scotland and Ireland; Scandinavia and the Baltics: Denmark, Finland, Norway, and Latvia; Western Europe: Austria, Germany, Luxembourg, and the Netherlands; Eastern Europe: Czech Republic and Hungary; Southern Europe: Greece, Malta, and Spain; North America: Canada, United States; South America: Brazil; Eastern Asia: the Republic of Korea; Oceania: Australia.

Table 2 shows the distribution of MDR *S*. Typhimurium presented as 2-year time-bands. Figure 3 depicts the general trend, with countries divided into 9 different regions. In 1992 15% of all *S*. Typhimurium isolates were MDR; by 2001, this percentage had increased 3-fold to 42%. Once again, large variations occurred between countries and within countries from year to year. In most European countries and North America, MDR *S*. Typhimurium was common and, with the exception of the United Kingdom and Ireland, multidrug resistance increased from the mid-1990s to the end of the study period. For example, in 2001 multidrug resistance ranged from 22% (Greece) to 72% (Ireland). Only limited data were available from Brazil, the Caribbean region, South Africa, and the Republic of Korea. However, in these regions multidrug resistance was far less common, ranging from zero in the Caribbean region to 19% in the Republic of Korea. In Australia, multidrug resistance remained low throughout the period, with 1.0% of strains in 1997 and 3.6% in 2001.

MDR DT104 was a frequent subtype of MDR *S*. Typhimurium in most of the countries. In 1992, between 11% (Germany) and 77% (Scotland) of the MDR strains were DT104; in 2001 this ranged from 22% (Australia) to 94% (the Netherlands). Overall, the proportion of MDR DT104 of all DT104 has remained fairly stable; 84% of DT104 were classified as MDR in 2001, compared with 72% in 1992. Among MDR DT104, the classical penta-resistant phenotype, R-type ACSSuT, was by far the most common phenotype throughout the period. It was found in 99% of MDR DT104 strains in 1992 and in 94% of such strains in 2001 (data not shown).

**Figure 4:**
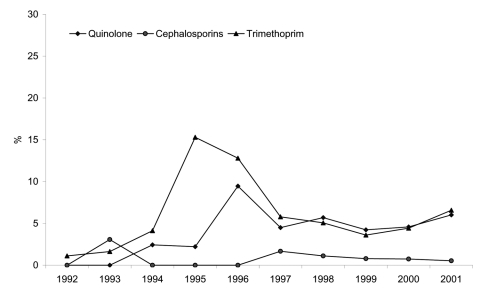
Proportion of multidrug-resistant *Salmonella enterica* serovar Typhimurium with additional resistance to quinolones, cephalosporin, or trimethoprim, 1992–2001.

Finally, we looked at trends in development of additional resistance in the classical penta-resistant phenotype (R-type ACSSuT), with focus on 3 clinically important antimicrobial drug classes: quinolones, cephalosporins, and trimethoprim. Both quinolone and trimethoprim resistance increased in MDR DT104 throughout the study period. In 1992, nalidixic acid susceptibility testing was performed on a total of 194 MDR DT104 isolates in 4 different countries; no resistant isolates were found. In 2001, nalidixic acid susceptibility testing of 1,812 MDR DT104 strains in 11 countries identified 109 (6.0%) resistant strains. Similarly, trimethoprim resistance was found in 1.2% of the 180 MDR DT104 strains tested in 1992, but in 6.6% of 1,855 MDR DT104 strains tested in 2001. Cephalosporin-resistant MDR DT104 remained rare, with only 0.5% resistant strains in 2001, and no clear trend observable. Figure 4 shows the overall trend of resistance to quinolone, trimethoprim, and cephalosporins from 1992 to 2001. The increase in quinolone resistance seen in 1996 and in 1998 was caused by a general increase in quinolone-resistant MDR DT104 in Scotland and an outbreak of quinolone-resistant MDR DT104 in Denmark (14), respectively. The increase in trimethoprim resistance from 1995 to 1996 was also caused by a general increase in trimethoprim-resistant MDR DT104 in Scotland.

## Discussion

The present survey was conducted to gain a better understanding of the global impact of DT104 and MDR *S*. Typhimurium, given the severity of illness than can result from infection with *S*. Typhimurium and MDR strains in particular. The survey's primary findings are that during the period 1992–2001, the total number of MDR *S*. Typhimurium and *S*. Typhimurium DT104 cases increased, while that of other types of *S*. Typhimurium decreased. The total number of nontyphoidal *Salmonella* and *S*. Typhimurium cases also decreased. However, these general findings mask large differences in regional trends. The collected data also may not always accurately describe the real national incidence or be directly comparable between countries.

The total number of isolates of nontyphoidal salmonellae registered at the national level decreased from 1992 to 2001. This result may be biased because of changes in surveillance practices. Since the survey indicated that surveillance systems generally improved throughout the study period, this trend is most likely correct, however. Concurrent with this decrease, an overall decrease in the number of *S*. Typhimurium isolates was observed, thus keeping the proportion of *S*. Typhimurium cases among total *Salmonella* cases constant. The decrease was primarily seen in Europe and North America, whereas Australia and New Zealand saw an increase in both the number of cases of *S*. Typhimurium and of the total number of nontyphoidal *Salmonella* cases. In Germany the total number of nontyphoidal *Salmonella* cases is available for the whole country, but for administrative reasons data on serotypes, antimicrobial susceptibility, and phage types are based only on results from the new federal states of Germany (formerly East Germany) and the city of Berlin. However, German studies have shown that serotype distribution and drug resistance are comparable between former West and East Germany (32) (W. Rabsch, pers. comm.).

MDR *S*. Typhimurium has increased in the past decades in almost all the regions covered in this study. Most of this increase is due to the concurrent upsurge of DT104, whereas other phage types, such as U302, DT120, DT12, and DT193, were reported to play a smaller role in this development. A high proportion of MDR *S*. Typhimurium was primarily observed in Europe and North America. Because of the low coverage of participating countries in Asia, South America, and Africa, the situation in these areas was difficult to assess. Both Australia and New Zealand, however, reported high incidence of *S*. Typhimurium, but MDR strains of *S*. Typhimurium and DT104 were largely absent. The isolated increase in MDR *S*. Typhimurium in Australia in 2001 can be explained by a large outbreak of MDR DT104 associated with consumption of *halva* (dessert made of sesame seeds) (33). That DT104 has not spread markedly in Australia and New Zealand may be explained both by geography and the very strict food and livestock import restrictions in force in these countries, which prevent any large-scale introduction and spread of foreign *Salmonella* types in the food animal production chain (34).

Although high and generally increasing levels of MDR *S*. Typhimurium were observed in Europe, the United Kingdom presents a special case. It and Germany had an increase in DT104 in the beginning of the 1990s, before most other countries (5,35). In fact, DT104 was first isolated in the United Kingdom in the early 1980s, years before it was isolated in other countries (5). In the United Kingdom, the incidence of DT104 peaked in 1996 and has since declined ([5] and this study). Possible explanations for this finding include the management of bovine spongiform encephalitis in the United Kingdom, associated general improvements in farm hygiene, and an overall decline in cattle production (36).

Although multidrug-resistance and DT104 were closely linked, large country-specific differences were seen, even between neighboring countries. Most pronounced was the difference between Germany and the Netherlands in 2001, where 64% and 94%, respectively, of MDR isolates were DT104. Such differences probably reflect both real differences and biases resulting from, for instance, different laboratory reporting practices. For example, some of the countries in this survey (New Zealand, Norway, and the United States) performed antimicrobial susceptibility tests on all their DT104 isolates but only on a subset of non-DT104 strains.

In Australia and in Scandinavia, where the incidence of domestically acquired salmonellosis is generally low, many cases appeared to be imported. In a recent study of Australian DT104 isolates, 37% were associated with travel abroad, particularly to Southeast Asia (D. Lightfoot, pers. comm.). Complete data on travel association of MDR *S*. Typhimurium cases were available for Norway and Finland and showed that most MDR *S*. Typhimurium patients were infected abroad. In Sweden, where information on DT104 but not MDR was available, most DT104 patients were infected abroad.

A special issue concerns the possibility of acquisition, with time, of resistance traits additional to the classical penta-resistant pattern. Of particular concern is the additional acquisition of quinolone resistance by MDR DT104, since fluoroquinolones are often the drugs of first choice when treating severe salmonellosis. Our data indicate that the prevalence of quinolone resistance has increased. As mentioned, several studies have now shown that multidrug resistance and quinolone resistance may be associated with particular adverse health effects. When seen in the light of the ability of DT104 to spread and establish itself in a large variety of food animal lines (cattle, pigs, poultry), the increase in the number of MDR *S*. Typhimurium strains that include quinolone resistance becomes particularly problematic. Quinolone, and in particular the fluoroquinolones, have been part of human medicine since the 1980s, resulting in no or very limited resistance in salmonellae. It was not until the license of fluoroquinolones for food animal production in the early 1990s that resistant *Salmonella* strains emerged (37). The use of fluoroquinolones for food production animals should therefore be discontinued or at least severely restricted as quickly as possible.

This survey has several important limitations. First, it was limited to countries with relatively sophisticated surveillance systems in place, since only countries performing phage typing or resistance testing in addition to serotyping were eligible. This meant that Asia, Africa, and South America were barely covered. Therefore, the survey contains no representative data on the situation in these regions. WHO Global Salm-Surv seeks to enhance the capacity of countries to provide such data. Second, the collection of isolates at the national level is likely to vary from country to country, depending on a number of factors such as sampling frequency at the local level, availability of laboratory reagents, and the degree to which isolates and results are forwarded to the national level. Local practice, the priority given to foodborne illnesses, and financial factors will influence on how often a physician will request a fecal sample. Furthermore, many countries stated that strains and information were not always routinely collected centrally, while in some of the countries strains were never forwarded from certain local laboratories. For these reasons, care should be taken in the interpretation of results of this survey; in particular when comparing incidence rates between countries. However, these limitations are inherent to surveillance in general and do not apply only to this survey. Cross-checking these survey data with available published surveillance data showed them to be in line with each other in the United States (38), the Netherlands (39), and Denmark (40).

In summary, on a global scale, only a small number of countries perform antimicrobial susceptibility testing or phage typing, although the number of countries doing so more than doubled throughout the study period. Despite its limitations, the survey showed that the incidence of both MDR *S*. Typhimurium and MDR *S*. Typhimurium DT104 increased markedly worldwide during the 1990s, although the problem has primarily affected Europe and North America. Of special concern is the increasing incidence of quinolone-resistant *S*. Typhimurium. The survey implies that MDR *S*. Typhimurium poses a serious and increasing public health problem in large parts of the world. Surveillance and control programs such as the Global Salm-Surv international network recently launched by WHO should therefore be reinforced.

## References

[1] Chalker RB, Blaser MJ. A review of human salmonellosis: III. Magnitude of *Salmonella* infection in the United States. Rev Infect Dis. 1988;10:111–24. 10.1093/clinids/10.1.111 2832925

[2] Tauxe R. *Salmonella* Enteritis and *Salmonella* Typhimurium DT104. Successful subtypes in the modern world. In: Emerging infections. Washington: ASM Press; 1999. p. 37–52.

[3] Gomez TM, Motarjemi Y, Miyagawa S, Kaferstein FK, Stohr K. Foodborne salmonellosis. World Health Stat Q. 1997;50:81–9. 9282390

[4] Swartz MN. Human diseases caused by foodborne pathogens of animal origin. Clin Infect Dis. 2002;34(Suppl 3):S111–22. 10.1086/340248 11988881

[5] Threlfall EJ, Ward LR, Frost JA, Willshaw GA. Spread of resistance from food animals to man—the UK experience. Acta Vet Scand Suppl. 2000;93:63–8. 10822859

[6] Rodrigues DC, Tauxe RV, Rowe B. International increase in *Salmonella* enteritidis: a new pandemic? Epidemiol Infect. 1990;105:21–7. 10.1017/S0950268800047609 2200698 PMC2271793

[7] Mead PS, Slutsker L, Dietz V, McCaig LF, Bresee JS, Shapiro C, Food-related illness and death in the United States. Emerg Infect Dis. 1999;5:607–25. 10.3201/eid0505.990502 10511517 PMC2627714

[8] Helms M, Vastrup P, Gerner-Smidt P, Mølbak K. Short and long term mortality associated with foodborne bacterial gastrointestinal infections: registry based study. BMJ. 2003;326:357. 10.1136/bmj.326.7385.357 12586666 PMC148890

[9] Martin LJ, Fyfe M, Dore K, Buxton JA, Pollari F, Henry B, Increased burden of illness associated with antimicrobial-resistant *Salmonella enterica* serotype Typhimurium infections. J Infect Dis. 2004;189:377–84. 10.1086/381270 14745694

[10] Helms M, Simonsen J, Mølbak K. Quinolone resistance is associated with increased risk of invasive illness and death in *Salmonella* Typhimurium infection. J Infect Dis. 2004;190:1652–4. 10.1086/424570 15478071

[11] Helms M, Vastrup P, Gerner-Smidt P, Mølbak K. Excess mortality associated with antimicrobial drug-resistant *Salmonella* Typhimurium. Emerg Infect Dis. 2002;8:490–5. 11996684 10.3201/eid0805.010267 PMC2732497

[12] Holmberg SD, Wells JG, Cohen ML. Animal-to-man transmission of antimicrobial-resistant Salmonella: investigations of U.S. outbreaks, 1971–1983. Science. 1984;225:833–5. 10.1126/science.6382605 6382605

[13] Boswell TC, Coleman DJ, Purser NJ, Cobb RA. Development of quinolone resistance in *Salmonella*: failure to prevent splenic abscess [letter]. J Infect. 1997;34:86–7. 10.1016/S0163-4453(97)80019-9 9120335

[14] Mølbak K, Baggesen DL, Aarestrup FM, Ebbesen JM, Engberg J, Frydendahl K, An outbreak of multidrug-resistant, quinolone-resistant *Salmonella enterica* serotype Typhimurium DT104. N Engl J Med. 1999;341:1420–5. 10.1056/NEJM199911043411902 10547404

[15] Varma J, Molbak K, Barrett TJ, Beebe JL, Jones TF, Rabatsky-Ehr T, Antimicrobial-resistant non-typhoidal salmonella is associated with excess bloodstream infections and hospitalizations. J Infect Dis. 2005;191:554–61. 10.1086/427263 15655779

[16] Cohen ML, Tauxe RV. Drug-resistant Salmonella in the United States: an epidemiologic perspective. Science. 1986;234:964–9. 10.1126/science.3535069 3535069

[17] Angulo FJ, Johnson KR, Tauxe RV, Cohen ML. Origins and consequences of antimicrobial-resistant nontyphoidal Salmonella: implications for the use of fluoroquinolones in food animals. Microb Drug Resist. 2000;6:77–83. 10.1089/mdr.2000.6.77 10868811

[18] Threlfall EJ, Rowe B, Ward LR. A comparison of multiple drug resistance in salmonellas from humans and food animals in England and Wales, 1981 and 1990. Epidemiol Infect. 1993;111:189–97. 10.1017/S0950268800056892 8405147 PMC2271397

[19] Ward LR, Threlfall EJ, Rowe B. Multiple drug resistance in salmonellae in England and Wales: a comparison between 1981 and 1988. J Clin Pathol. 1990;43:563–6. 10.1136/jcp.43.7.563 2199536 PMC502581

[20] Witte W. Medical consequences of antibiotic use in agriculture. Science. 1998;279:996–7. 10.1126/science.279.5353.996 9490487

[21] Glynn MK, Bopp C, Dewitt W, Dabney P, Mokhtar M, Angulo FJ. Emergence of multidrug-resistant *Salmonella enterica* serotype Typhimurium DT104 infections in the United States. N Engl J Med. 1998;338:1333–9. 10.1056/NEJM199805073381901 9571252

[22] Rabsch W, Tschape H, Baumler AJ. Non-typhoidal salmonellosis: emerging problems. Microbes Infect. 2001;3:237–47. 10.1016/S1286-4579(01)01375-2 11358718

[23] Threlfall EJ. Epidemic *Salmonella* Typhimurium DT 104-a truly international multiresistant clone. J Antimicrob Chemother. 2000;46:7–10. 10.1093/jac/46.1.7 10882682

[24] Herikstad H, Motarjemi Y, Tauxe RV. *Salmonella* surveillance: a global survey of public health serotyping. Epidemiol Infect. 2002;129:1–8. 10.1017/S0950268802006842 12211575 PMC2869853

[25] Global Salm-Surv [homepage on the Internet]. World Health Organization. 2003 [cited 2005 Mar 31]. Available from www.who.int/salmsurv/en

[26] Fisher IS. The Enter-Net international surveillance network–how it works. Eurosurveillance. 1999;4:52–5. 12631902 10.2807/esm.04.05.00073-en

[27] Standard country or area codes for statistical use [monograph on the Internet]. United Nations Statistics Division. 2004 Mar 8 [cited 2005 Mar 31]. Available from http://unstats.un.org/unsd/methods/m49/m49.htm

[28] Anderson ES, Ward LR, Saxe MJ, de Sa JD. Bacteriophage-typing designations of *Salmonella* Typhimurium. J Hyg (Lond). 1977;78:297–300. 10.1017/S0022172400056187 321679 PMC2129838

[29] Rabsch W. Laborgestützte epidemiologische Analysen–Methodenspektrum und epidemiologische Bewertung. Klassische epidemiologische Laboratoriumsmethoden. In: Kühn H, Tschape H, editors. Salmonellosen des Menschen—Epidemiologische und ätiologische Aspekte. Munich: Medizin Verlag; 1996. p. 118–34.

[30] van Duijkeren E, Wannet WJ, Houwers DJ, van Pelt W. Antimicrobial susceptibilities of *Salmonella* strains isolated from humans, cattle, pigs, and chickens in the Netherlands from 1984 to 2001. J Clin Microbiol. 2003;41:3574–8. 10.1128/JCM.41.8.3574-3578.2003 12904357 PMC179820

[31] van Duijkeren E, Wannet WJ, Houwers DJ, van Pelt W. Serotype and phage type distribution of *Salmonella* strains isolated from humans, cattle, pigs, and chickens in the Netherlands from 1984 to 2001. J Clin Microbiol. 2002;40:3980–5. 10.1128/JCM.40.11.3980-3985.2002 12409362 PMC139702

[32] Kuehn H. Vorkommen und epidemische Verbreitung. In: Kühn H, Tschäpe H, editors. Salmonellosen des Menschen–Epidemiologische und ätiologische Aspekte. Munich: Medizin Verlag; 1996. p. 19–35.

[33] Fisher I, Andersson Y, De Jong B, O'Grady KA, Powling J. International outbreak of *Salmonella* Typhimurium DT104–update from Enter-Net. Eurosurveillance Weekly. 2001;5.

[34] Crump JA, Murdoch DR, Baker MG. Emerging infectious diseases in an island ecosystem: the New Zealand perspective. Emerg Infect Dis. 2001;7:767–72. 10.3201/eid0705.010501 11747690 PMC2631882

[35] Prager R, Liesegang A, Rabsch W, Gericke B, Thiel W, Voigt W, Clonal relationship of *Salmonella enterica* serovar Typhimurium phage type DT104 in Germany and Austria. Zentralbl Bakteriol. 1999;289:399–414. 10.1016/S0934-8840(99)80081-4 10603659

[36] Threlfall EJ, Ward LR, Skinner JA, Graham A. Antimicrobial drug resistance in non-typhoidal salmonellas from humans in England and Wales in 1999: decrease in multiple resistance in *Salmonella enterica* serotypes Typhimurium, Virchow, and Hadar. Microb Drug Resist. 2000;6:319–25. 10.1089/mdr.2000.6.319 11272261

[37] Threlfall EJ, Ward LR, Rowe B. Resistance to ciprofloxacin in non-typhoidal salmonellas from humans in England and Wales-the current situation. Clin Microbiol Infect. 1999;5:130–4. 10.1111/j.1469-0691.1999.tb00525.x 11856236

[38] Salmonella Surveillance CDC. annual summary, 2001. Atlanta: Centers for Disease Control and Prevention; 2002.

[39] van Pelt W, de Wit MA, Wannet WJ, Ligtvoet EJ, Widdowson MA, van Duynhoven YT. Laboratory surveillance of bacterial gastroenteric pathogens in The Netherlands, 1991–2001. Epidemiol Infect. 2003;130:431–41. 12825727 PMC2869979

[40] Annual report on zoonoses in Denmark 2001 [monograph on the Internet]. Ministry of Food, Agriculture and Fisheries. 2002 [cited 2005 Mar 31]. Available from http://www.dfvf.dk/files/filer/zoonosecentret/publikationer/ annual%20report/annual_report_2001_fra_datagraf.pdf

